# Differential Effect on Hippocampal Synaptic Facilitation by the Presynaptic Protein Mover

**DOI:** 10.3389/fnsyn.2019.00030

**Published:** 2019-11-15

**Authors:** Julio S. Viotti, Thomas Dresbach

**Affiliations:** Institute of Anatomy and Embryology, University Medical Center Göttingen, Georg-August University of Göttingen, Göttingen, Germany

**Keywords:** synaptic transmission, short-term plasticity, hippocampus, CA3, mossy fiber, presynapse

## Abstract

Neurotransmitter release relies on an evolutionarily conserved presynaptic machinery. Nonetheless, some proteins occur in certain species and synapses, and are absent in others, indicating that they may have modulatory roles. How such proteins expand the power or versatility of the core release machinery is unclear. The presynaptic protein Mover/TPRGL/SVAP30 is heterogeneously expressed among synapses of the rodent brain, suggesting that it may add special functions to subtypes of presynaptic terminals. Mover is a synaptic vesicle-attached phosphoprotein that binds to Calmodulin and the active zone scaffolding protein Bassoon. Here we use a Mover knockout mouse line to investigate the role of Mover in the hippocampal mossy fiber (MF) to CA3 pyramidal cell synapse and Schaffer collateral to CA1. While Schaffer collateral synapses were unchanged by the knockout, the MFs showed strongly increased facilitation. The effect of Mover knockout in facilitation was both calcium- and age-dependent, having a stronger effect at higher calcium concentrations and in younger animals. Increasing cyclic adenosine monophosphate (cAMP) levels by forskolin equally potentiated both wildtype and knockout MF synapses, but occluded the increased facilitation observed in the knockout. These discoveries suggest that Mover has distinct roles at different synapses. At MF terminals, it acts to constrain the extent of presynaptic facilitation.

## Introduction

The molecular machinery mediating neurotransmitter release is strongly conserved throughout evolution: Ca^2+^ triggers exocytosis of neurotransmitter from synaptic vesicles (SVs) in less than a millisecond by binding to Synaptotagmin, which together with Complexins activates a core fusion machinery composed of SNAREs and SV proteins (Südhof, 2013). These events are confined to specialized sites of the presynaptic plasma membrane, called active zones, by a network of proteins including RIMs, RIM binding proteins, Munc13s, α-liprins and CAST/ERC proteins. One of the ways by which these scaffolding molecules act is that RIMs recruit both calcium channels and Munc13s, which make SVs tethered at the active zone fusion competent (Südhof, 2012; Imig et al., 2014; Acuna et al., 2016). The physical and functional interactions of all these proteins represent an evolutionarily conserved machinery mediating regulated neurotransmitter release at active zones. The evolutionarily conserved Ca^2+^ binding proteins Calmodulin and Synaptotagmin-7 further refine the machinery by regulating presynaptic plasticity such as short-term depression and short-term enhancement to transmitter release (Junge et al., 2004; Jackman et al., 2016).

Piccolo/Aczonin and Bassoon are particularly large active zone scaffolding molecules, consisting of approximately 5000 and 4000 amino acids, respectively (Cases-Langhoff et al., 1996; tom Dieck et al., 1998; Wang et al., 1999). Piccolo is evolutionarily related to RIM (Bruckner et al., 2012) and shares 10 Piccolo-Bassoon homology regions with Bassoon (tom Dieck et al., 1998; Gundelfinger et al., 2016). Piccolo and Bassoon do not seem to be essential for the process of transmitter release (Hallermann et al., 2010; Mukherjee et al., 2010; Mendoza Schulz et al., 2014; Butola et al., 2017; Parthier et al., 2018), but maintain synaptic integrity by reducing the degradation of presynaptic molecules by proteasomes and autophagy (Waites et al., 2013; Okerlund et al., 2017). As scaffolding proteins that appear very early at nascent active zones and bind a large number of proteins they may also act to recruit regulatory molecules to active zones (Fejtova and Gundelfinger, 2006; Schoch and Gundelfinger, 2006; Gundelfinger et al., 2016).

In a yeast-2-hybrid assay we identified the SV phospho-protein Mover – also called SVAP-30 and TPRGL (Burré et al., 2006; Antonini et al., 2008) – as a binding partner for Bassoon (Kremer et al., 2007; Ahmed et al., 2013). Mover is a small, 266 amino acid, protein that does not appear to have orthologs in the nematode *Caenorhabditis elegans* and the fruit fly Drosophila, suggesting that it is not required for the basic functions of the transmitter release machinery. In the rodent brain, its distribution is remarkably heterogeneous. For example, inhibitory synapses in the hippocampal CA3 region lack Mover, while excitatory synapses in the same region contain Mover (Kremer et al., 2007). Quantitative analysis revealed that the levels of Mover relative to the number of SVs vary among synapses throughout the brain (Wallrafen and Dresbach, 2018). These observations suggest that Mover may perform regulatory functions at certain synapses. To test how the absence of Mover affects synaptic transmission, we investigated two different hippocampal synapses. We assumed that synapse function would not be abolished, but a modulatory role would emerge. We found that the absence of Mover affects short-term plasticity in the hippocampal CA3 but not in CA1. We show that this effect is age- and Ca^2+^-dependent, and interacts with the cyclic adenosine monophosphate (cAMP) pathway in the mossy fiber (MF) synapses.

## Materials and Methods

### KO Generation, Genotyping, and Confirmation

All animal experiments were performed in accordance with the guidelines for the welfare of experimental animals issued by the State Government of Lower Saxony, Germany. All mice (*Mus musculus*) were bred and maintained at the central animal facility of the University Medical Center, Göttingen. Embryonic stem cells (129/Ola) harboring the recombined Mover locus were generated by PolyGene (Switzerland), and injected into blastocysts. Mice with the targeted locus were crossed with a Flp deleter line to remove the Neo cassette. The resulting conditional knockout mice were crossed with mice expressing Cre recombinase under the E2A promoter to generate a global Mover knockout (Akula et al., 2019). Knockout (KO) animals were compared to their wildtype (WT) littermates.

Mice were routinely genotyped by PCR using genomic DNA extracted from tail biopsies using a DNA extraction kit from NextTec (Germany). For amplification the following oligonucleotide primers were used: forward primer P1 (5′-CCAATCACAAGGCGAACGAG-3′); forward primer P2 (5′-CATTCAGTGGGACAAGCAGA-3′); reverse primer P3 (5′-CTTGGATCAGGAGAGCCTTG-3′). The PCR reaction was carried out for 40 cycles with denaturation at 95°C for 30 s, annealing at 56°C for 1 min, and extension at 72°C for 1 min. WT and KO animals were identified by the presence of a specific 867 bp and a 697 bp band, respectively. Initially, the KO was verified by purifying and sequencing the 697 bp band.

For immunohistochemical confirmation of the knockout of Mover, mice were perfused with 4% paraformaldehyde and had their hippocampi sectioned with 50 μm thickness. Commercially available anti-Mover and anti-Synaptophysin antibodies (Synaptic Systems, Germany) were used in combination with Cy2 and Cy5 secondary antibodies (Dianova, Germany). Stainings were visualized by epifluorescence through an Observer Z1 inverted microscope (Zeiss, Germany).

### Slice Preparation and Electrophysiology

Acute transverse hippocampal slices 400 μm thick were prepared from male and female 18–22 days old mice unless otherwise noted. Hippocampi were isolated and cut in a Thermo Scientific HM650V vibratome in a cutting solution containing (in mM): 215 sucrose, 2.5 KCl, 20 glucose, 26 NaHCO_3_, 1.6 NaH_2_PO_4_, 1 CaCl_2_, 4 MgCl_2_, and 4 MgSO_4_. After sectioning, the slices were incubated for 30 min in a solution comprised of 50% cutting solution and 50% recording solution, which contained (in mM): 124 NaCl, 2.5 KCl, 26 NaHCO_3_, 1 NaH_2_PO_4_, 2.5 CaCl_2_, 1.3 MgSO_4_, and 10 glucose; 300 mOsm/kg. Recording solution for whole-cell patch clamp recordings, was comprised of (in mM): 119 NaCl, 2.5 KCl, 26 NaHCO_3_, 1 NaH_2_PO_4_, 4 CaCl_2_, 4 MgSO_4_, and 10 glucose; 300 mOsm/kg. After the aforementioned 30 min incubation, the mixed solution was changed to 100% recording solution. After changing of solution, slices were incubated for at least 60 min at room temperature. All solutions were continuously gassed with carbogen (95% O_2_, 5% CO_2_) and were recorded at 27 ± 0.2^*o*^C.

Excitatory post synaptic potentials (EPSPs) and receptor excitatory postsynaptic currents (EPSCs) were recorded using a HEKA EPC-10 amplifier connected to a chlorided silver wire in a borosilicate glass pipette. Recording pipettes had 0.5–1.5 MΩ pipette resistance and were filled with 1 M NaCl for extracellular recordings, and 2.0–4.0 MΩ for whole-cell recordings with a solution containing (in mM): 123 Cs-gluconate, 8 NaCl, 10 HEPES, 10 Glucose, 10 BAPTA, 5 ATP-Mg, 0.4 GTP-Na; 300 mOsm/kg, pH 7.2. Stimulation of axons was delivered through a patch-type pipette connected to either a Model A395 linear stimulus isolator (World Precision Instruments) or a Model DS3 isolated current stimulator (Digitimer).

Schaffer collateral responses were recorded at the stratum radiatum of the CA1, with the stimulation electrode placed rostral to the recording electrode. Mossy fiber field EPSPs (fEPSPs) were recorded in the stratum lucidum of the hippocampus, whereas whole-cell recordings were performed by patching CA3 pyramidal cells. In both cases the MFs were stimulated at the border between the dentate gyrus and the hilus. At the end of each MF experiment, the group II metabotropic glutamate receptor agonist DCG-IV (2S,20R,30R-2-[20,30-dicarboxycyclo-propyl] glycine) was applied to the bath (1 μM) to selectively block MF responses (Kamiya et al., 1996). Recordings in which responses did not reduce by at least 80% were excluded from the analysis. For whole-cell recordings 100 μM picrotoxin was added to the solution to avoid inhibitory transmission. For each sample, every recording was repeated at least three times and traces were averaged. Data was sampled at 20 or 50 kHz and low-pass filtered at 2.9 kHz.

### Experimental Design and Statistical Analysis

Electrophysiological data were analyzed using custom-written procedures in Igor Pro 6.3 (Wavemetrics). Statistical significance was tested using GraphPad Prism 7.04 (GraphPad Software). Two-way ANOVA tests were used to analyze high-frequency trains of stimulation. Extra sum-of-squares *F* test was used to test differences between curve fits. This statistical method was chosen due to its robustness in comparing two nested models and returning a *p*-value in response to whether one curve adequately fits both data sets or whether two different curves better describe the data (Motulsky and Christopoulos, 2003). For all other tests two-tailed Student’s unpaired *T* test was used. For every experiment 3 or more animals were used. Results are reported as mean ± SEM whereas “n” refers to the number of slices recorded. Stimulation artifacts were digitally removed from electrophysiological traces for clarity.

## Results

**FIGURE 1:**
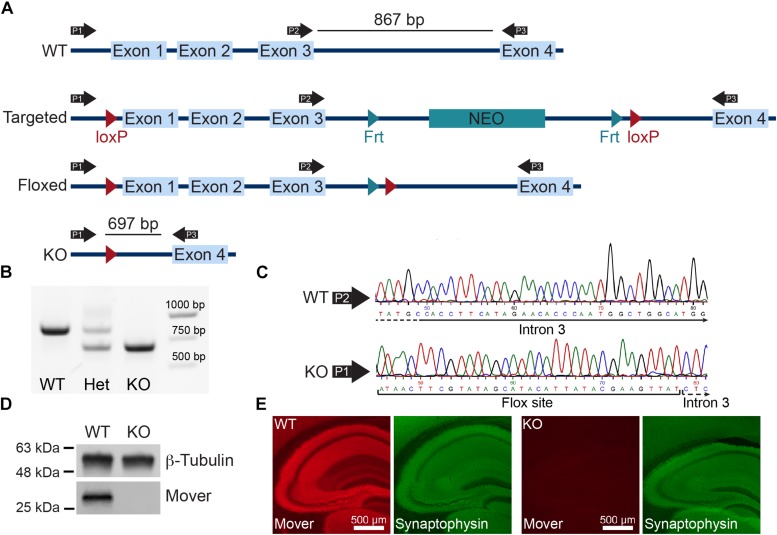
Global knockout of Mover. **(A)** Gene targeting strategy for Mover KO mice. **(B)** Results of the PCR used for genotyping. Primers P1, P2, and P3 shown in panel **(A)** were always used in the same reaction. When a WT and a KO allele were present, P1 and P3 produce a 697 bp band, P2 and P3 produce a 867 bp band (lane “Het”). When only WT alleles are present the primers produce only the 867 bp band (lane “WT”), when only KO alleles are present the primers produce only the 697 (lane “KO”). **(C)** Example of sequencing results for WT (top) and KO (bottom). Examples shown start from nucleotide 45 from sequencing result and show a part of intron 3 using the primer P2 for WT and the flox site followed by intron 3 in KO, showing the absence of exons 1–3. **(D)** Western blot of lysates from dissected hippocampi from WT (left) and KO mice (right) probed for β-Tubulin and Mover. **(E)** Immunofluorescence of WT (left) and KO (right) mouse brain sections stained for Mover and Synaptophysin.

To obtain a global knockout of Mover we bred Mover conditional knockout mice generated in the lab (Akula et al., 2019) with E2A-Cre mice. The E2A promoter drives Cre expression in the early mouse embryo, thus excising Mover in all cells from early embryonic stages on. The entire Mover gene consists of less than 4000 base pairs, including four exons and three introns (Figure 1A). We verified the expected excision of Mover exons 1, 2, and 3 by PCR (Figure 1B), and by sequencing the PCR product (Figure 1C). Western blotting revealed that Mover was not detected in hippocampal lysates from Mover knockout mice (Figure 1D). Likewise, there was no Mover immunofluorescence in sections of the hippocampus from Mover knockout mice (Figure 1E).

### Mover Knockout Affects Mossy Fiber but Not Schaffer Collateral Synaptic Transmission

**FIGURE 2:**
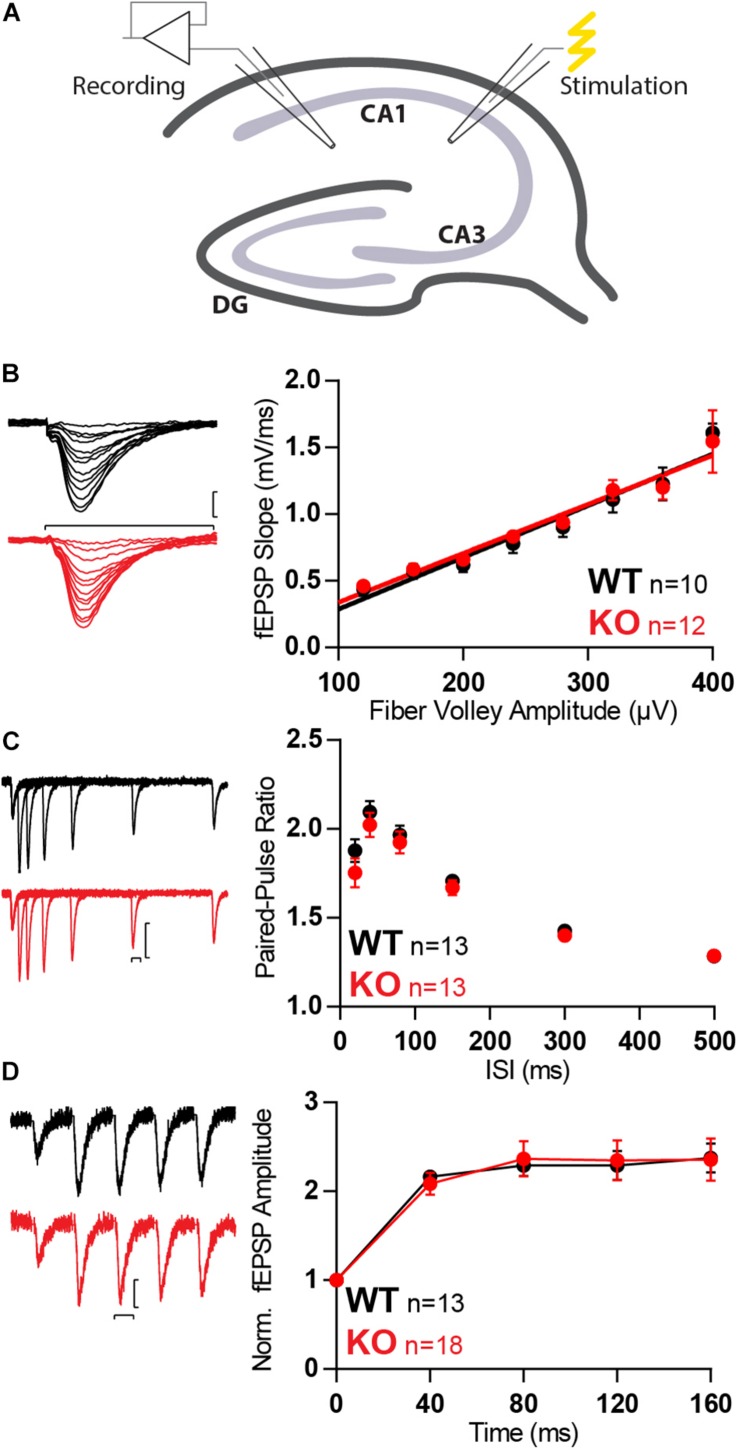
Synaptic transmission is unchanged in SC in absence of Mover. **(A)** Diagram representing stimulation of Schaffer collaterals and extracellular recording at stratum radiatum of the CA1. **(B)** Example traces (left) and quantification (right) of fEPSP slopes versus fiber volley amplitude recorded at increasing stimulation strengths depicts an input–output relationship unchanged by the absence of Mover. **(C)** Paired-pulse ratios recorded from fEPSP amplitudes across varying inter-stimulus intervals show no difference between WT and KO. **(D)** Normalized responses to a 25 Hz train of stimulation showed unchanged facilitation in KO MF when compared to WT. Scale bars: vertical = 250 μV, horizontal = 20 ms. Error bars indicate SEM. WT *n* = 10–13; KO *n* = 12–18.

The absence of Mover in inhibitory synapses of the hippocampus (Wallrafen and Dresbach, 2018), together with its layered structure, makes it an ideal target to study the effect of Mover knockout in glutamatergic transmission. Recording of fEPSPs in the hippocampal CA1 with stimulation of the Schaffer collaterals (SC, Figure 2A) with increasing stimulation strength allows for an assessment of synaptic strength based on input–output curves. Input–output curves showed no difference in synaptic strength between WT and KO mice (Figure 2B).

To further probe changes in presynaptic transmission we evaluated short-term plasticity with three different protocols: paired-pulse facilitation with two pulses given at different inter-stimulus intervals (ISIs; Figure 2C), burst-induced facilitation with a 25 Hz train consisting of five stimuli (Figure 2D). In all protocols no differences between WT and KO were detected.

**FIGURE 3:**
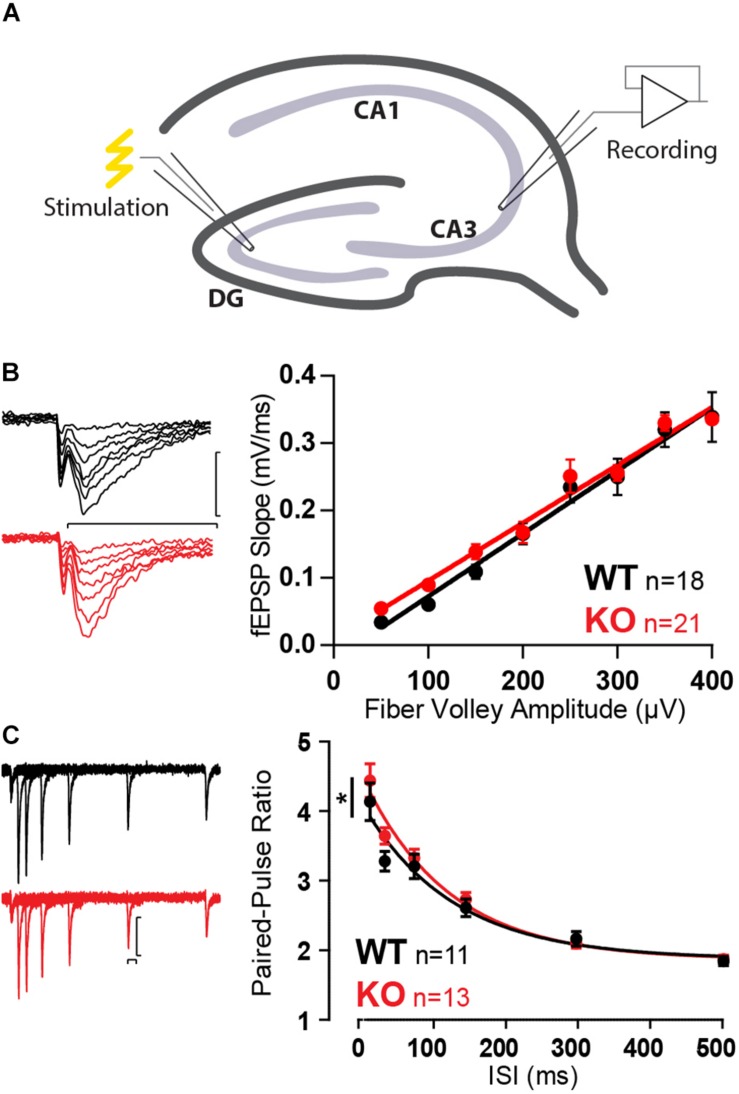
Increased paired-pulse ratio in MF in absence of Mover. **(A)** Diagram representing stimulation of mossy fibers and extracellular recording in the stratum lucidum of the CA3. **(B)** Example traces (left) and quantification (right) of fEPSP slopes versus fiber volley amplitude recorded at increasing stimulation strengths depicts an input–output relationship unchanged by the absence of Mover. **(C)** Paired-pulse ratios recorded from fEPSP amplitudes across varying inter-stimulus intervals reveal increased facilitation in KO when compared to WT. Scale bars: vertical = 250 μV, horizontal = 20 ms. Error bars indicate SEM. MF WT *n* = 11–18; KO *n* = 13–22. ^∗^*p* < 0.05.

With no apparent differences in SC, we moved to investigate the CA3, more specifically the CA3 pyramidal cell inputs from MFs (Figure 3A). Mover was first observed to have high expression in the stratum lucidum, where MF form boutons with CA3 pyramidal cells (Kremer et al., 2007). However, we have recently shown that Mover is also strongly present in the stratum radiatum, where SC synapses are present (Wallrafen and Dresbach, 2018). MF boutons have very low release probability with particularly strong short-term plasticity effects (Nicoll and Schmitz, 2005) and would more easily reveal any possible changes in synaptic function in the absence of Mover.

Similarly to SC, input–output curves were unchanged by the absence of Mover (Figure 3B). However, a first assessment of the short-term plasticity parameters of MF KO already indicated a change in the absence of Mover: paired-pulse facilitation was slightly increased in KO synapses (Figure 3C), most pronouncedly at 40 ms ISI (*p* = 0.039, Extra-sum-of-squares *F* test).

**FIGURE 4:**
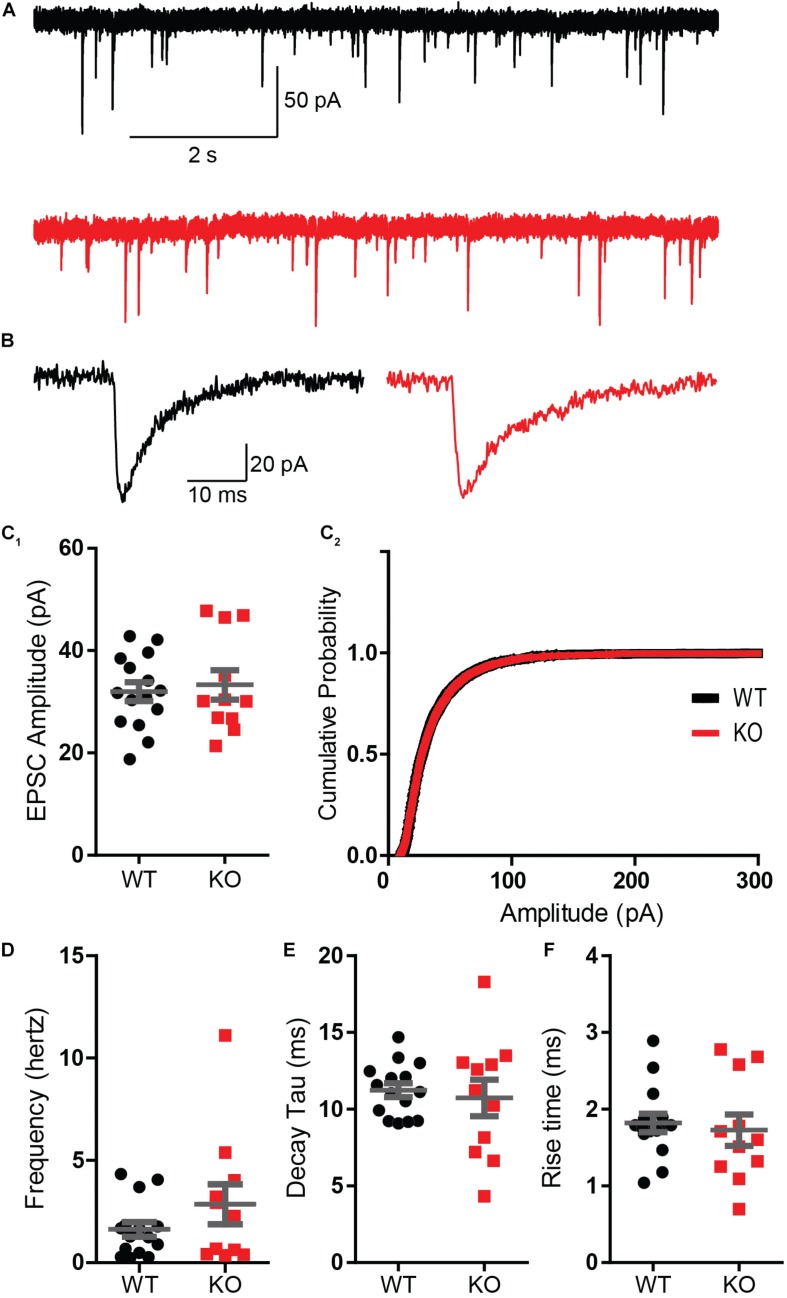
Absence of Mover does not interfere with miniature EPSC parameters in CA3 pyramidal cells. **(A)** Representative traces from WT (gray) and KO (red) CA3 pyramidal cells under presence of 1 μM TTX. **(B)** Representative miniature EPSC waveform from traces in A. **(C)** Amplitudes of miniature EPSC events were unchanged in their average amplitude **(C_1_)** and in their cumulative probability **(C_2_)**. **(D)** Frequency of events was not changed by the absence of Mover. Miniature EPSC kinetics, namely the time constant of decay **(E)** and the 10–90 rise time **(F)**, showed no difference between WT and KO. Error bars indicate SEM. WT *n* = 15; KO *n* = 11.

These results were surprising considering that a previous report using whole-cell recordings found a reduced paired-pulse ratio in a knockdown of Mover at the calyx of Held (Körber et al., 2015). Therefore, to verify our finding we employed whole-cell patch clamp recordings from CA3 pyramidal cells. We initially characterized spontaneous activity onto the pyramidal cells in the presence of 1 μM tetrodotoxin (TTX) and 100 μM of the GABA receptor blocker picrotoxin, so that recordings comprised of only miniature EPSCs. Amplitude and frequency of miniature EPSCs were unchanged in the KO (Figures 4A–D). The kinetics of transmission, namely rise time and decay, were also comparable between WT and KO (Figures 4E,F).

With absence of changes in spontaneous synaptic activity in the CA3 pyramidal cells we set out to verify the change in facilitation observed in extracellular recordings using intracellular recordings. These recordings were also done in the presence of 100 μM picrotoxin to abolish possible biases introduced by inhibitory transmission. Because the CA3 is a highly auto-associative area (Hablitz, 1984; Traub et al., 1993) we took extra precautions to prevent polysynaptic inputs by blocking α-amino-3-5-methyl-4-isoxazolepropionic acid (AMPA) receptors with 10 μM of 2,3-dihydroxy-6-nitro-7-sulfamoylbenzo[f]quinoxaline-2,3-dione (NBQX). Therefore, the responses comprised of *N*-methyl-D-aspartate receptor EPSCs (NMDA-EPSCs).

**FIGURE 5:**
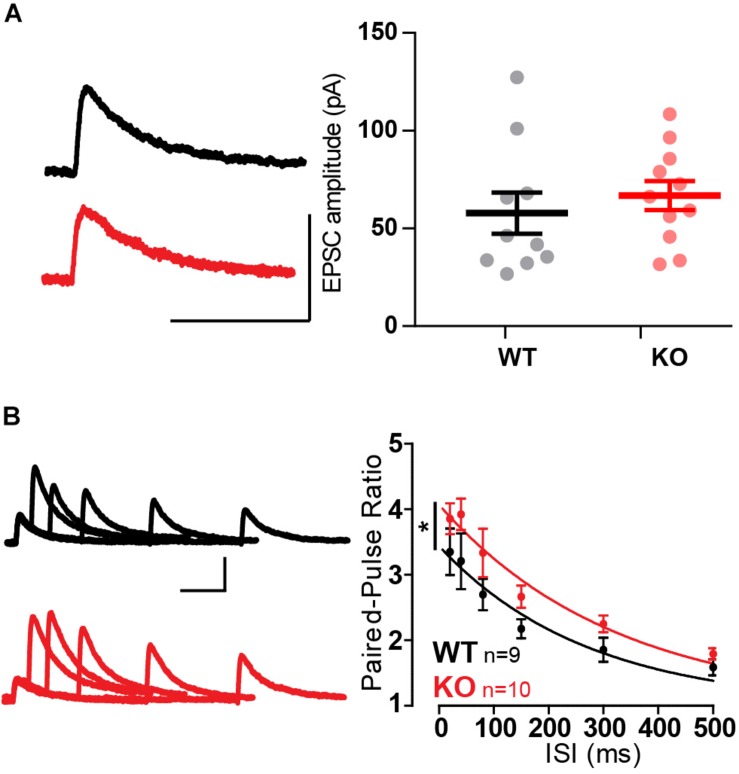
Whole-cell recordings confirm increased paired-pulse ratio in MF-CA3 pyramidal cells in Mover KO. **(A)** Example traces and quantification of single evoked NMDA EPSCs had similar amplitude between WT and KO. **(B)** Paired-pulse ratios recorded from NMDA EPSC amplitudes across varying inter-stimulus intervals show increased ratios in KO pyramidal cells. Scale bars: vertical = 100 pA, horizontal = 100 ms. Error bars indicate SEM. WT *n* = 9; KO *n* = 10. ^∗^*p* < 0.05.

The amplitude of single evoked NMDA-EPSCs did not vary between WT and KO (Figure 5A). Confirming the previous observation in extracellular recordings and adding to it, paired-pulse facilitation was increased across all ISIs tested (Figure 5B, *p* = 0.016, Extra-sum-of-squares *F* test). This confirmed the change in short-term plasticity observed with extracellular recordings.

### Mover Strongly Affects Release Upon Repetitive Stimulation

One of the hallmarks of MF is the ability to strongly facilitate, not only during a paired-pulse protocol, but also during repetitive stimulation under a vast range of frequencies (Nicoll and Schmitz, 2005). We therefore proceeded to analyze the effect of Mover deletion on responses upon repetitive stimulation, which could make the effects of the deletion even more prominent.

**FIGURE 6:**
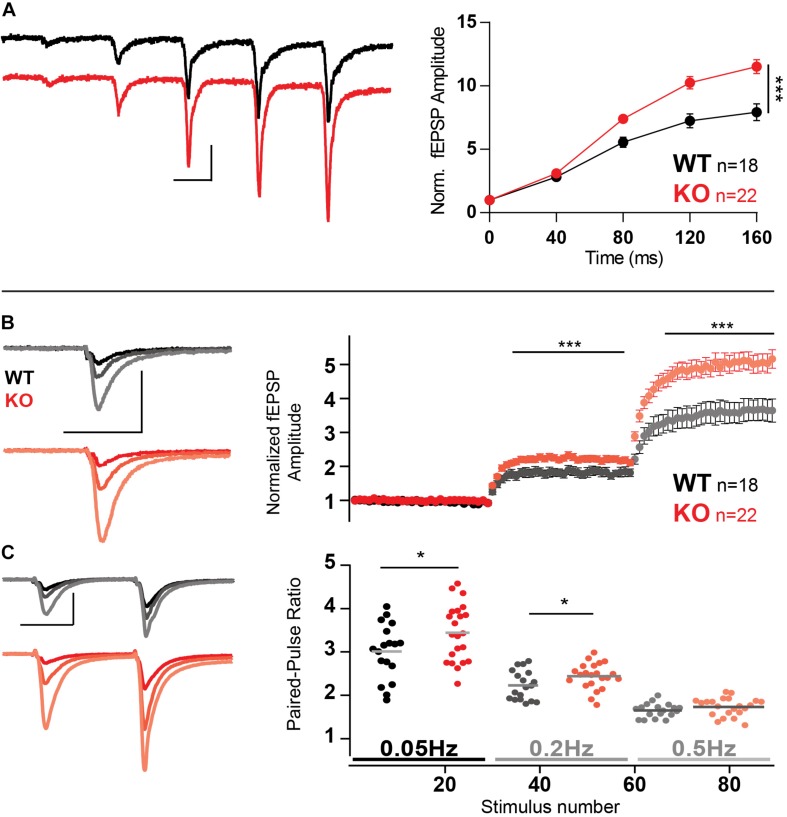
Increased facilitation in MF in the absence of Mover. **(A)** Example traces and normalized responses to 25 Hz trains of stimulation showed increased facilitation in KO MF when compared to WT. **(B)** Normalized responses to stimuli delivered at 0.05, 0.2, and 0.5 Hz reveal increased facilitation in KO MF. Responses were normalized to the amplitude of the first fEPSP. **(C)** Paired-pulse ratio with an inter-stimulus interval of 40 ms using the same stimulation frequencies as above (0.05, 0.2, and 0.5 Hz, see text). Scale bars: vertical = 500 μV, horizontal = 20 ms. Error bars indicate SEM. SC WT *n* = 10–13; SC KO *n* = 12–18; MF WT *n* = 18; KO *n* = 22. ^∗^*p* < 0.05; ^∗∗∗^*p* < 0.001.

Trains of five stimuli at 25 Hz with extracellular recordings elicited a strong facilitation in both WT and KO MF. However, KO MF had even stronger facilitation than WT (*p* = 0.0004, two-way ANOVA), reaching more than a 10-fold increase in fEPSP amplitude (Figure 6A).

An increase in synaptic transmission in response to increasing stimulation frequencies, even at low frequencies, is a hallmark of MF facilitation, often referred as frequency facilitation (Nicoll and Schmitz, 2005). Using low-frequency stimulation led to similar enhanced facilitation of KO responses: change from frequency of stimulation from 0.05 to 0.2 Hz led to KO responses 30% stronger than WT (Figure 6B, *p* = 0.0007). Further increasing the frequency of stimulation to 0.5 Hz led to KO responses to facilitate 40% more than WT (*p* < 0.0001).

Furthermore, we probed the paired-pulse facilitation elicited by stimulation of an ISI of 40 ms, repeated with a waiting time of either 20 s (0.05 Hz), 5 s (0.2 Hz), or 2 s (0.5 Hz), i.e., the frequencies described in the previous experiment. Because both frequency facilitation and paired-pulse ratio depend on the residual calcium inside the presynaptic terminal we expected that the two would interact negatively, i.e., as frequency facilitation rises, paired-pulse ratio would decrease; and Mover should still affect paired-pulse ratio. Indeed, as expected, paired-pulse ratios were smaller at higher frequencies and the absence of Mover increase paired-pulse ratio on frequencies 0.05 (Figure 6C, *p* = 0.042) and 0.2 Hz (*p* = 0.046).

Because of this recent evidence of its influence on MF short-term facilitation and previous evidence of its influence in calcium-sensitivity of release (Körber et al., 2015), we decided to investigate whether there is a calcium-dependency of Mover effects on synaptic release. And since residual calcium is changed in MF with age (Mori-Kawakami et al., 2003) we decided to investigate the effect of the KO in older animals.

### Mover Affects MF Facilitation Under High Calcium Conditions

The extent of facilitation in MF is known to reduce with age (Mori-Kawakami et al., 2003). Hence, to further explore the effect of the absence of Mover we analyzed the effect of MF stimulation in older mice (8-week old) with different extracellular calcium concentrations. The change in calcium concentration was compensated with magnesium, to keep the concentration of divalent ions constant.

**FIGURE 7:**
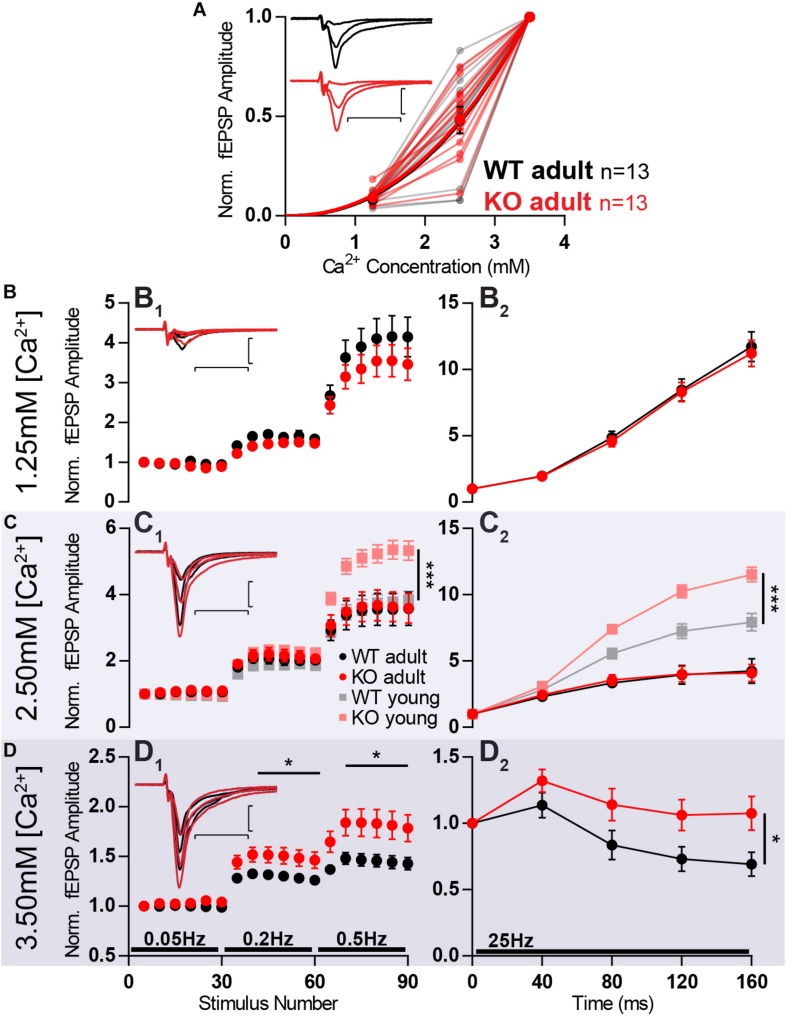
Increased facilitation in KO is age- and calcium-dependent. MF of 8-week old animals KO (KO adult) has stronger facilitation than WT (WT adult) only in high extracellular calcium concentration. **(A)** Increasing calcium concentration leads to similar baseline responses in WT and KO. Responses were normalized to fEPSP amplitudes at 3.5 mM Ca^2+^. **(B–D)** Short-term plasticity at different extracellular calcium concentrations. **(B_1_)** Normalized responses to stimuli delivered at 0.05, 0.2, and 0.5 Hz at 1.25 mM extracellular calcium. **(B_2_)** Normalized responses to a 25 Hz train of stimulation at 1.25 mM extracellular calcium. **(C_1_)** Normalized responses to stimuli delivered at 0.05, 0.2, and 0.5 Hz at 2.5 mM extracellular calcium, in four different conditions: 8-week old WT (adult), 8-week old KO (adult), 3-week old WT (WT young), and 3-week old KO (KO young). The last two conditions are the same dataset as presented in Figures 6A,B. **(C_2_)** Normalized responses to a 25 Hz train of stimulation at 2.5 mM extracellular calcium. **(D_1_)** Normalized responses to stimuli delivered at 0.05, 0.2, and 0.5 Hz at 3.5 mM extracellular calcium. **(D_2_)** Normalized responses to a 25 Hz train of stimulation at 3.5 mM extracellular calcium. **(B_1_,C_1_,D_1_)** Each dot represents the average response to five consecutive stimuli. **(B–D)** Responses were normalized to the amplitude of the first fEPSP. (**Insets**) Representative traces from WT (*black*) and KO (*red*) hippocampi. Scale bars: vertical = 1 mV, horizontal = 10 ms. Error bars indicate SEM. WT adult *n* = 13; KO adult *n* = 13. ^∗^*p* < 0.05; ^∗∗∗^*p* < 0.001.

Firstly, increasing the concentration of calcium from 1.25 to 2.5 mM and further to 3.5 mM led to an increase in fEPSP amplitude. This increase was similar when comparing WT and KO responses (Figure 7A).

When testing the previously used short-term plasticity protocols, at 1.25 mM Ca^2+^ the extent of facilitation did not differ between WT and KO anymore (Figure 7B). Surprisingly, after increasing Ca^2+^ to 2.5 mM, WT and KO continued to facilitate to the same extent (Figure 7C), whereas the same calcium concentration in 3 week-old animals promoted a stronger facilitation in MF KOs (Figures 6A,B). This reveals an age-dependency of the effect that Mover has on facilitation.

When further increasing Ca^2+^ concentration to 3.5 mM, the difference in the extent of facilitation between KO and WT becomes obvious again for both high-frequency (Figure 7D_2_; *p* = 0.04) and low-frequency facilitation (Figure 7D_1_; 0.05 Hz vs. 0.2 Hz: *p* = 0.04; 0.05 Hz vs. 0.5 Hz: *p* = 0.03). The KO responses facilitate more than WT, corroborating what was observed in younger animals and the idea that Mover acts in a calcium-dependent manner.

### Forskolin Occludes the Boost in Facilitation Observed in KO

Synaptic plasticity in MF is known to be strongly tied to intracellular levels of cAMP and is, therefore, subject to regulation by forskolin (Weisskopf et al., 1994). Hence, since both forskolin and Mover strongly affect plasticity in MF we decided to investigate if Mover acts in the cAMP pathway.

**FIGURE 8:**
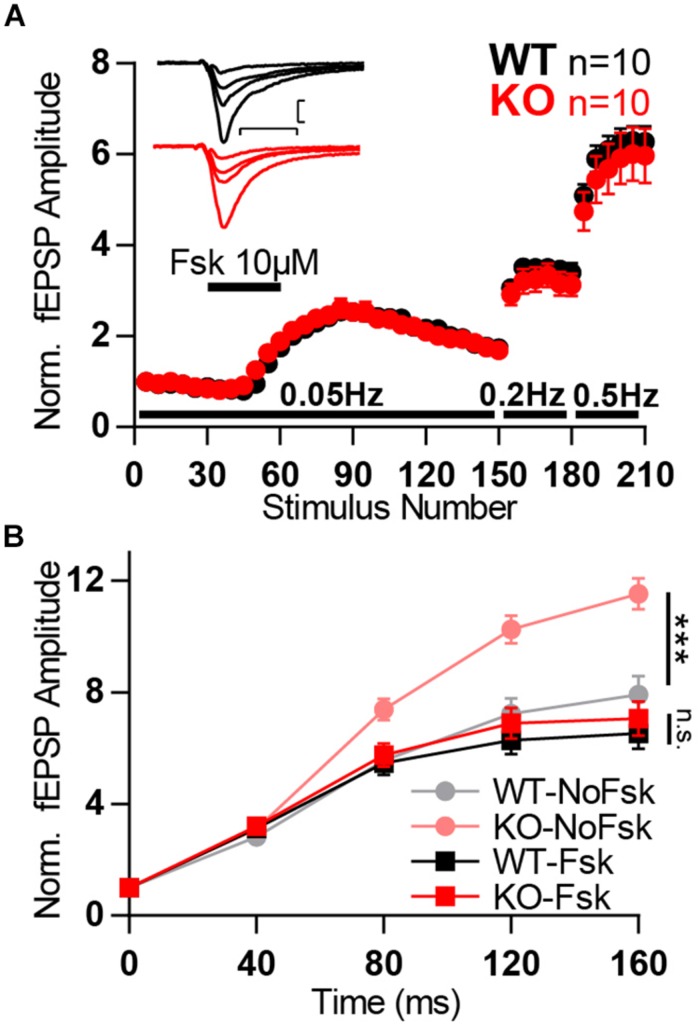
Forskolin potentiation occlude KO boost in facilitation. **(A)** Normalized MF fEPSP amplitudes during the time course of experiment in which forskolin (*fsk*, 10 μM) is applied for 10 min and frequency of stimulation is changed from 0.05 to 0.2 and further to 0.5 Hz. Each data point corresponds to the average response to five consecutive stimuli. (Inset) Representative traces from WT (*black*) and KO (*red*) hippocampi. **(B)** Normalized MF responses to a 25 Hz train of stimulation in four different conditions: WT without forskolin application (WT-NoFsk), KO without forskolin application (KO-NoFsk), WT after forskolin potentiation (WT-Fsk), and KO after forskolin potentiation (KO-Fsk). The first two conditions are the same dataset as present in Figure 6B_._ Responses were normalized to the amplitude of the first response. Scale bars: vertical = 200 μV, horizontal = 10 ms; WT *n* = 10; KO *n* = 10. Error bars indicate SEM. n.s.: not significant; ^∗∗∗^*p* < 0.001.

We anticipated that, if Mover participates in this pathway and is involved in the potentiation caused by forskolin, we would observe changes in the degree of potentiation between WT and KO. However, application of forskolin led to a similar potentiation of WT and KO fEPSPs, suggesting that Mover is not necessary forskolin-driven potentiation. On the other hand, after forskolin potentiation we observed a lack of difference between WT and KO low- and high-frequency facilitation (Figures 8A,B). This lack of a KO effect in facilitation after forskolin potentiation contrasts with the increased facilitation we observed in the absence of forskolin (Figures 6A,B, also in Figure 8B for comparison). Therefore, potentiation by forskolin occluded the increase in facilitation observed in the KO. These results suggest that Mover interacts with the cAMP pathway in MF, dampening facilitation in situations of high activity.

## Discussion

Mover is a synaptic vesicle protein with a remarkably heterogenous expression pattern in the rodent brain (Wallrafen and Dresbach, 2018). In addition, *C. elegans* and Drosophila lack Mover related genes. Thus, Mover is not essential for synaptic transmission. Instead, it may modulate neurotransmitter release at certain synapses, contributing to synaptic heterogeneity. To test whether Mover has a role in transmitter release we analyzed a mouse-line lacking Mover. We found that the knockout of Mover affects short-term synaptic plasticity in the hippocampal MFs but not in the downstream synapses, i.e., SC. Paired-pulse ratio and responses to train of stimulation led to stronger facilitation in MF terminals in the absence of Mover. In particular, frequency facilitation, a hallmark of presynaptic plasticity at MF terminals (Nicoll and Schmitz, 2005), was strongly increased in Mover knockout mice. This increased facilitation was stronger in younger animals and in situations of high calcium concentration, and was occluded by increasing cAMP levels.

### Mover Has Synapse-Specific Effects on Neurotransmitter Release

The increase in paired-pulse facilitation in KO MF can be explained by a reduction in synaptic vesicle release probability, because of the inverse relationship between the two (Zucker and Regehr, 2002). The change in MF contrasts with the absence of changes in release in KO SC. Curiously, despite the lack of the effect of the KO in SC, Mover concentration in WT mice is known to be higher in the area of SC synapses than that of MF synapses (Wallrafen and Dresbach, 2018), so the effect of Mover on synaptic release does not seem to scale with its concentration. To add to the heterogeneity of Mover function, a study in the calyx of Held described an increase in release probability in a knockdown of Mover in the calyx of Held (Körber et al., 2015). The contrasting results between the calyx of Held and MF could arise from the different approach (knockdown vs. knockout) or from the different species (rat vs. mouse). However, when seen in combination with the lack of changes in SC, a hypothesis arises: Mover has synapse-specific effects. The synapse-specific way in which Mover seems to regulate transmission could add heterogeneity to the ubiquitous functions of the transmitter release machinery. In addition, we speculate that at certain synapses, i.e., at MF and the calyx of Held, the differential action of Mover could have a common effect in constraining synaptic strength. MF transmission relies heavily on facilitation for efficient information transfer and is, therefore, considered a “conditional detonator” (Vyleta et al., 2016). We have shown that Mover constrains facilitation in this synapse, possibly keeping detonation within physiological range. On the other hand, Mover reduces the amplitude of the first response to a train of stimuli and subsequent depression in a synapse where this initial response is vital for transmission of fast and reliable auditory information, the calyx of Held (Englitz et al., 2009; Körber et al., 2015). Thus, in general, Mover could have the potential to act as a buffer for synaptic strength. Further experiments to test this in different synapses and to elucidate the mechanism of action of Mover could help prove or disprove such a hypothesis.

Such a differential effect on synaptic release in different synapses is not exclusive to Mover. For example, a similar synapse-specific effect on hippocampal synaptic transmission and plasticity was previously observed with the priming protein Munc13-2, where deletion of this protein led to increased facilitation and lower release probability in MF but not in SC (Breustedt et al., 2010). In the case of Munc13-2, the mechanism through which it affects neurotransmitter release is more straightforward since the Munc13 proteins are known to be vesicle priming factors (Augustin et al., 1999; Varoqueaux et al., 2002). Alternatively, Mover may affect facilitation directly – with or without affecting release probability – in a similar way like the calcium sensor protein Synaptotagmin 7 (Jackman et al., 2016). In this case, however, Mover would be acting as an inhibitor of facilitation, i.e., in the opposite direction compared to Synaptotagmin 7, which increases facilitation. In any case, Mover seems to be under the influence of Ca^2+^, as discussed below (see section “Ca^2+^ Dependency of Mover Action”).

### Calcium Dependency of MF Plasticity: Technical Considerations

Synaptic facilitation is largely dependent on free or bound residual Ca^2+^ in the presynaptic terminal (Zucker and Regehr, 2002; Jackman and Regehr, 2017). One exception to this is the activity-dependent release of polyamine blocks in AMPA receptors (Rozov and Burnashev, 1999), but it is not present in MF to CA3 pyramidal synapses (Toth et al., 2000). Facilitation at MF-CA3 synapse is closely correlated with the concentration of Ca^2+^ in the presynaptic terminal (Regehr et al., 1994) and happens over a wide range of frequencies (Salin et al., 1996), which we also observed in our experiments.

Different mechanisms have been proposed to explain the relationship between Ca^2+^ and facilitation at this synapse. First, the presence of calcium buffers coupled with a loose coupling between Ca^2+^ channels and Ca^2+^ sensors (Blatow et al., 2003; Vyleta and Jonas, 2014) could act in a way that repeated stimulation could saturate the buffers and allow for a stronger presence of available Ca^2+^ to act on the release sensor. Another proposed mechanism is the presence of a second Ca^2+^ sensor, a “facilitation sensor,” with high affinity and slow kinetics, which would remain bound to calcium after the action potential-evoked calcium transient. In Jackman et al. (2016) proposed that this sensor is Synaptotagmin 7 as the KO of Synaptotagmin 7 shows a dramatic reduction on the degree of MF facilitation. Other mechanisms that could underlie facilitation at MF-CA3 synapse include distinct contribution from different Ca^2+^ channel types (Chamberland et al., 2017), modulation by adenosine receptors (Moore et al., 2003; Gundlfinger et al., 2007a; Klausnitzer and Manahan-Vaughan, 2008), by Kainate receptors (Lauri et al., 2001; Schmitz et al., 2001; Breustedt and Schmitz, 2004) often in connection with increased Ca^2+^ influx or Ca^2+^ release from internal stores (Kamiya et al., 2002; Lauri et al., 2003; Scott and Rusakov, 2006; Scott et al., 2008) and the CaM and/or adenylyl cyclase pathways (Salin et al., 1996; Wang et al., 2003; Rodríguez-Moreno and Sihra, 2004; Andrade-Talavera et al., 2012).

The mechanisms that affect facilitation in response to high-frequency stimulation and low-frequency stimulation (frequency facilitation) greatly overlap. It has been previously described that both phenomena act on residual calcium and occlude each other (Salin et al., 1996). As in Salin et al. (1996), our work shows that increase of frequency facilitation led to a reduction in paired-pulse facilitation (Figure 6), suggesting that both processes operate under a common mechanism. Admittedly, due to the long-lasting nature of frequency facilitation, and different modulation between the two time ranges (Gundlfinger et al., 2007b), the involvement of intermediary biochemical processes that are independent of residual Ca^2+^ have been suggested (Nicoll and Schmitz, 2005; Sihra and Rodríguez-Moreno, 2013) possibly by acting in the CaM and cAMP pathways (Salin et al., 1996; Wang et al., 2003; Rodríguez-Moreno and Sihra, 2004; Andrade-Talavera et al., 2012). Nevertheless, manipulations affecting frequency facilitation but not facilitation in the millisecond range still need to be performed to decisively show a mechanistic difference between the two different time scales.

In order to study how Mover is affecting facilitation and its calcium-dependency we have used different extracellular Ca^2+^ concentrations, which influence the Ca^2+^ influx into the synaptic terminal and changes release probability. The increase in Ca^2+^ was compensated by a decrease in extracellular Mg^2+^ in the opposite direction. This way, the concentration of divalent ions was kept constant. This minimizes the possible voltage shifts that could occur due to changes in surface charge screening. Nevertheless, we cannot rule out that shifts could have occurred since Ca^2+^ produces a stronger shift in surface potential than Mg^2+^ (Hille, 2001). Other mechanisms through which changing extracellular Ca^2+^ concentrations can lead to changes in excitability include its binding to voltage-gated channels, blocking channel pores, through a G-protein coupled receptor called calcium-sensing receptor (CaSR) and through a calcium- and voltage-dependent cation channel called calcium homeostasis modulator 1 (Armstrong and Cota, 1990, 1991; Santarelli et al., 2007; Ma et al., 2012; Jones and Smith, 2016). In every case increased Ca^2+^ concentration leads to decreased excitability. On the other hand, the decrease of Mg^2+^ can also lead to consequences in neuronal transmission. Most notably, the reduction of Mg^2+^ concentration would lead to a reduced block on NMDA receptors (Nowak et al., 1984). The activation of NMDA receptors by glutamate in this situation would lead to stronger EPSCs with a slower decay. While the aforementioned effects may occur in our approach, we applied changes in Ca^2+^ and Mg^2+^ concentrations equally to both WT and KO samples. This way, even though excitability and synaptic responses would be altered when the Ca^2+^/Mg^2+^ ratio is changed they should be comparable between WT and KO and should not interfere with the analysis done here.

It is also important to note that CA3 pyramidal cells form an auto-associative network through associational recurrent axonal collaterals that target other CA3 pyramidal cells (Hablitz, 1984; Traub et al., 1993; Nicoll and Schmitz, 2005). Such organization can lead to the situation where the cell being recorded shows responses, not to a MF input, but from the synaptic release from another pyramidal cell, which was activated by the stimulation. Therefore, di- or poly-synaptic contamination, or even contamination from direct stimulation of association/commissural fibers is common in CA3 recordings. We took the following precautions to ensure monosynaptic MF-CA3 pyramidal cell responses. Firstly, recordings were succeeded by application of DCG-IV, which selectively blocks MF responses (Kamiya et al., 1996). Therefore, recordings in which responses did not reduce by 80% were excluded from the analysis. Secondly, in whole-cell recordings NBQX was applied to the bath so that only NMDA receptor responses would occur. Because NMDA receptors are blocked by Mg^2+^ at resting potential polysynaptic responses are unlikely to occur (Weisskopf and Nicoll, 1995; Nicoll and Schmitz, 2005). Furthermore, the use of 4 mM Ca^2+^ and Mg^2+^ during whole-cell recordings further ensures pure monosynaptic responses due to three factors: increased block of NMDA receptors at resting membrane potential, reduced release probability and hyperpolarization of neurons due to surface charge screening (Nowak et al., 1984; Hille, 2001). Such strategies are commonly used (Salin et al., 1996; Kwon and Castillo, 2008a, b; Kaeser-Woo et al., 2013) to ensure absence of contamination in recordings and better quality of recordings.

### Ca^2+^ Dependency of Mover Action

How does Mover interact with calcium signaling or the release machinery? The amino acid structure of Mover does not reveal any canonical calcium binding domain (Kremer et al., 2007). Here we observe an occlusion of the KO effect after activation of adenylyl cyclase by forskolin, which suggests that Mover and adenylyl cyclase – or its product, cAMP – act in the same pathway. Activation of adenylyl cyclase has been shown to increase neurotransmitter release in MF (Weisskopf et al., 1994; Tzounopoulos et al., 1998; Rodríguez-Moreno and Sihra, 2004). cAMP has been shown to influence release in MF through its direct targets Epac2 (exchange protein directly activated by cAMP) and protein kinase A (Weisskopf et al., 1994; Rodríguez-Moreno and Sihra, 2004). One proposed way through which synaptic activity increases cAMP levels is by the activation of adenylyl cyclases 1 and 8 by CaM (Villacres et al., 1998; Wang et al., 2003; Sihra and Rodríguez-Moreno, 2013). Mover could participate in this pathway via its known CaM binding property (Körber et al., 2015; Akula et al., 2019). Further experiments would be necessary to test this hypothesis.

The participation of Mover in a Ca^2+^-sensing pathway is corroborated by the observation that Mover affects MF plasticity differently under different extracellular calcium concentrations (Figure 7). This differential response indicates that there is a calcium-dependency of Mover action: it only influenced release in higher calcium concentrations.

The Ca^2+^-dynamics in MF also seem to change with age. The knockout of Mover also did not show an effect on short-term plasticity in older mice under the same experimental conditions as juvenile mice (Figure 7). When comparing 3- and 9-week old mice Mori-Kawakami et al. (2003) show reduced paired-pulse ratios and frequency-dependent facilitation in older mice. This was attributed to a lower availability of residual Ca^2+^. This lower Ca^2+^ concentration, in addition to the calcium-dependency of the effect of Mover explains the smaller effect of the KO in older animals. It is also possible to assume that homeostatic plasticity could have played a stronger role in the older mice and, therefore, reduced the consequences of the absence of Mover. Nonetheless, the exact mechanism through which Mover acts to, most likely indirectly, sense intracellular Ca^2+^ levels and influence release will be the object of future studies.

## Data Availability Statement

The datasets generated for this study are available on request to the corresponding author.

## Ethics Statement

The animal study was reviewed and approved by the Tierschutzkommission der Universitätsmedizin Göttingen.

## Author Contributions

JV performed the research, analyzed the data, designed the experiments, and wrote the manuscript. TD designed the experiments and wrote the manuscript.

## Conflict of Interest

The authors declare that the research was conducted in the absence of any commercial or financial relationships that could be construed as a potential conflict of interest.
